# Genomics of Otitis Media (OM): Molecular Genetics Approaches to Characterize Disease Pathophysiology

**DOI:** 10.3389/fgene.2020.00313

**Published:** 2020-04-23

**Authors:** Arnaud P. J. Giese, Saadat Ali, Amal Isaiah, Ishrat Aziz, Saima Riazuddin, Zubair M. Ahmed

**Affiliations:** ^1^Department of Otorhinolaryngology—Head and Neck Surgery, University of Maryland School of Medicine, Baltimore, MD, United States; ^2^The Institute of Biochemistry and Biotechnology, University of Veterinary and Animal Sciences, Lahore, Pakistan; ^3^Department of Biotechnology, Virtual University of Pakistan, Lahore, Pakistan

**Keywords:** otitis media (OM), omic, genetic, FUT, fucosyltransferase, A2ML1

## Abstract

Otitis media (OM) is an infective and inflammatory disorder known to be a major cause of hearing impairment across all age groups. Both acute and chronic OM result in substantial healthcare utilization related to antibiotic prescription and surgical procedures necessary for its management. Although several studies provided evidence of genetics playing a significant role in the susceptibility to OM, we had limited knowledge about the genes associated with OM until recently. Here we have summarized the known genetic factors that confer susceptibility to various forms of OM in mice and in humans and their genetic load, along with associated cellular signaling pathways. Spotlighted in this review are fucosyltransferase (FUT) enzymes, which have been implicated in the pathogenesis of OM. A comprehensive understanding of the functions of OM-associated genes may provide potential opportunities for its diagnosis and treatment.

## Introduction

Otitis media (OM) is defined as an infective and inflammatory disorder of the middle ear. While OM is associated with significant heterogeneity in clinical presentation, the broad types include acute otitis media (AOM), chronic suppurative otitis media (CSOM), and chronic otitis media with effusion (OME). Previous studies have shown that the pooled incidence of AOM is about 11% worldwide, with 51% of cases occurring in children under the age of five (Monasta et al., 2012). The recurrence of AOM may lead to OME, which has a worldwide incidence rate of up to 5% (Monasta et al., 2012). Both AOM and OM continue to be associated with healthcare utilization in the form of antibiotic therapy, physician and emergency room visits, and common surgical procedures such as tympanostomy tubes, although the advent of effective antimicrobial therapy has led to a substantial reduction in the burden of CSOM (Thomas et al., 2004). A smaller number of studies have also described an association between middle ear infections and speech and language deficits, emphasizing the role of OM in childhood development (Roberts et al., 2004).

The most common bacteria isolated from the middle ear of patients with AOM include *Streptococcus pneumoniae* and *Haemophilus influenzae*, although *Moraxella catarrhalis*, *Streptococcus pyogenes*, and *Staphylococcus aureus* are less frequently observed. In contrast, *Pseudomonas aeruginosa* and *S. aureus* are the most frequently observed pathogens in CSOM (Giebink and Canafax, 1991).

OM is a multifactorial disorder that may be attributed to a combination of etiologic factors including immunologic, genetic, environmental, and anatomic characteristics. Seasonal microbial susceptibility and Eustachian tube dysfunction are the commonly observed causes (Swanson and Hoecker, 1996; Fireman, 1997). It is well-known that viruses from the respiratory airways also play a crucial role in the pathogenesis of AOM (Nokso-Koivisto et al., 2015). Further, exposure to tobacco smoke, the use of a pacifier, and daycare attendance are among the risk factors for OM, while breastfeeding and pneumococcal vaccines have protective effects (Swanson and Hoecker, 1996; Lubianca Neto et al., 2006; Abrahams and Labbok, 2011; Norhayati et al., 2017).

Beyond environmental factors, genetic background also confers susceptibility to OM, although the disease mechanism is not fully understood. Several OM-associated genes, identified through studies in humans and in animal models, are known to play fundamental roles in diverse biological processes including (1) the development of the middle ear cleft and the Eustachian tube, (2) immune response, (3) bacterial adhesion and viral infection rate, (4) regulation of extracellular matrix, and (5) clearance of pathogens (see Tables 1, 2 for specific studies). In this review, we summarized the genomic variants and factors that have been reported in patients with various forms of OM. Early genetic association studies, mouse, mouse-to-man, human candidate, and genome-wide association studies that correlate OM and genetic variations are also briefly discussed. However, we particularly focused on the recent findings of the associations of A2ML1 and FUT enzymes with OM and offered our perspective on the potential disease mechanism that intuitively can lead to OM in individuals harboring variants of *FUT2*.

**TABLE 1:** Most common loci associated with otitis media in mouse studies.

**Locus**	**Protein**	**Protein function/phenotype in mouse mutant**	**OMIM**	**References**
*C3H/Hej*	*TLR4* mice strain	*TLR4* mice are prone to bacterial infection	603030	Mitchell et al., 1997
*Ccl3*	C-C motif chemokine 3	Downstream of TNF-mediated inflammation pathways	182283	Leichtle et al., 2010
*Cfb*	Complement factor B	*Streptococcus pneumoniae* induced increased gene expression of factor B of the alternative complement pathway and C3 in mouse middle ear epithelium	138470	Li et al., 2011
*Chd7*	Chromodomain-helicase-DNA-binding protein 7	OM with effusion	608892	Tian et al., 2012
*Eda*	Ectodysplasin-A	Otitis media, rhinitis, and nasopharyngitis	300451	Azar et al., 2016
*Edar*	Tumor necrosis factor receptor superfamily member EDAR	Otitis media, rhinitis, and nasopharyngitis	604095	Azar et al., 2016
*Enpp1*	Ectonucleotide pyrophosphatase/phosphodiesterase family member 1	OM with effusion in *Enpp1*^*asj*^ mutant mice	173335	Tian et al., 2016
*Ets1*	E26 transformation-specific 1	Craniofacial abnormalities, small middle ear cavity, short nasal bone, hearing impairment, otitis media, fusion of ossicles to the middle ear wall, and deformed stapes	164720	Carpinelli et al., 2015
*Evi1*	Ectotropic viral integration 1	Chronic suppurative OM with otorrhea	165215	Parkinson et al., 2006
*Eya4*	Eyes absent homolog 4	Abnormal middle ear cavity and Eustachian tube	603550	Depreux et al., 2008
*Fbxo11*	F-box only protein 11	Compound heterozygotes carrying both Jeff and Mutt alleles demonstrated a shortened face, reduced hearing, and OM	607871	Hardisty-Hughes et al., 2006
*Fli1*	Friend leukemia integration 1 transcription factor	Craniofacial abnormalities, small middle ear cavity, short nasal bone, hearing impairment, otitis media, fusion of ossicles to the middle ear wall, and deformed stapes	193067	Carpinelli et al., 2015
*Hbegf*	Heparin binding EGF- like growth factor	Mucosal epithelial hyperplasia	126150	Suzukawa et al., 2014
*Hif*	Hypoxia inducible factor	Hypoxia and signal abruptions	603348	Cheeseman et al., 2011
*Jnk1*	JNK1	C57BL/6 mice deficient in JNK1 exhibit enhanced mucosal thickening	601158	Yao et al., 2014
*Jnk2*	JNK2	JNK2-/- mice exhibit delayed mucosal hyperplasia, delayed recruitment of neutrophils, and failure of bacterial clearance	602896	Yao et al., 2014
*Lysozyme M*	Lysozyme M	*Lysozyme M* deficiency leads to an increased susceptibility to *Streptococcus pneumoniae*-induced OM	153450	Shimada et al., 2008
*Lmna*	Prelamin-A/C	Malformation and abnormal positioning of the Eustachian tube, accompanied by OM, were observed in all of the Lmna(Dhe/+) mutant mice	150330	Zhang et al., 2012
*MyD88*	Myeloid differentiation primary response protein MyD88	Delayed recruitment of neutrophils and macrophages	602170	Hernandez et al., 2008
*Mcp1/Ccl2*	C-C motif chemokine 2	MCP-1/CCL2 contributes to inner ear inflammation secondary to NTHi -induced OM	158105	Woo et al., 2010
*Math1*	Protein atonal homolog 1	Important for mucous cell differentiation	601461	Nakamura et al., 2013
*Mph1*	Sex comb of midleg	OM with effusion in the hearing-impaired Mcph1(tm1a) (/tm1a) mutant mice	300227	Chen et al., 2013
*Oxgr1*	2-Oxoglutarate receptor 1	COME	606922	Kerschner et al., 2013
*Pai1*	Plasminogen activator inhibitor 1	Bullae of PAI-1 mutant mice showed low levels of inflammation against NTHi at the early stage of OM	173360	Shin et al., 2014
*Pax9*	Paired box protein Pax-9	Expression reduced in Slc25a21(tm1a(KOMP)Wtsi) mutant mice; leads to inflammation of the middle ear	167416	Maguire et al., 2014
*Phex*	Phosphate-regulating neutral endopeptidase PHEX	Mutation in *Phex* gene predisposes the BALB/c-Phex(Hyp-Duk)/Y mice to OM	300550	Han et al., 2012
*Plg*	Plasminogen	Infiltration of neutrophils and macrophages as well as the presence of T and B cells in the middle ear mucosa	173350	Eriksson et al., 2006
*Spag6*	Sperm-associated antigen 6	*Spag6* mutant mice are prone to develop OM due to accumulation of fluid and mucus secondary to the ciliary dysfunction.	605730	Li et al., 2014
*Sh3pxd2b*	SH3 and PX domain-containing protein 2B	Sh3pxd2b(nee) mutant mice develop craniofacial dysmorphologies and OM changes in cilia and goblet cells of the middle ear mucosa in Sh3pxd2b(nee) mutant mice were observed	613293	Yang et al., 2011
*Slc25a21*	Solute carrier Family 25 member 21	Homozygosity for Slc25a21(tm1a(KOMP)Wtsi) results in mice exhibiting orofacial abnormalities, alterations in carpal and rugae structures, hearing impairment, and inflammation in the middle ear	607571	Maguire et al., 2014

**TABLE 2:** Most common loci associated with otitis media in human studies.

**Gene**	**Chr:**	**Protein**	**Function/pathway**	**Marker**	**Country**	**Sample size**	**Significance**	**Clinical outcome**	**References**
*A2ML1*	12	Alpha-2-macroglobulin-like protein 1 (A2ML1)	Peptidase inhibitor activity	c.2478_2485dupGG CTAAAT (p.Ser829Trpfs*9), p.Glu972*	Philippines	Familial (affected = 38, unaffected = 13)	LOD = 7.5	OM	Santos-Cortez et al., 2015
*ABO*	9	Histo-blood group ABO system transferase	Blood type	Type O: c.260insG(p.Val87_ Thr88fs*) Type A	Finland	214 probands	Type A: (OR = 2.14; 95% CI: 1.04–4.50; *p* = 0.03) type O: (OR = 0.33; 95% CI: 0.11–1.04; *p* = 0.04)	RAOM/COME A increases risk for COME c.260insG (p.Val87_Thr88fs*) variant and type O are protective against RAOM	Wiesen et al., 2019
*CD14*	5	Cluster of differentiation 14 (CD14)	Immune response, co-receptor of TRL4	rs2569190	Netherlands	ca = 74, co = 35	*p* = 0.004	AOM	Wiertsema et al., 2006a
*CPT1A*	11	Carnitine palmitoyl transferase type 1A (CPT1A)	Fatty acid oxidation	rs80356779	Alaska	ca = 291, co = 136	*p* < 0.001	OM	Gessner et al., 2013
*CX3CR1*	3	CX3C chemokine receptor 1	Binds to chemokine	rs3732378	USA	ca = 653	*p* = 0.038		Nokso-Koivisto et al., 2014
*FBXO11*	2	F-box only protein 1 (FBXO11)	Protein ubiquitination	rs10182633 rs12620679 rs12712997 rs13430439 rs2710163 rs33787 rs6713506 rs6728843 rs12712997	Australia	ca = 253, co = 866	*p* = 0.0009 *p* = 0.001 *p* = 0.0002 *p* = 0.0061 *p* = 0.0003 *p* = 6.9 × 10^–6^ *p* = 0.0074 *p* = 0.0061	AOM	Rye et al., 2011
				rs330787		ca = 434 families, co = 561	*p* = 0.009 *p* = 0.053	RAOM/COME	
*FCGR2A*	1	Fc gamma receptor 11a (FCGR2A)	Fc gamma receptor, immune response	rs1801274	Netherlands	ca = 383	*p* = 0.03	OM after PV	Wiertsema et al., 2006b
*FNDC1*	6	Fibronectin type III domain-containing protein 1 (FNDC1)	May be an activator of G protein signaling	rs2932989	European	825 cases and 7,936 control	*p*_*meta*_ = 2.15 × 10^–09^	AOM	van Ingen et al., 2016
*FUT2*	19	Galactoside 2-alpha-L-fucosyltransferase 2 (FUT2)	Creates H antigen, essential for the formation of ABO blood group antigens	rs1800022, rs601338, rs149356814, rs602662	Philippines, Pakistan, USA	1 Filipino consanguineous pedigree 609 multi-ethnic families and simplex case subjects with OM	LOD = 4.0	COME, AOM, OM	Santos-Cortez et al., 2018
*IFNG*	12	IFN γ	Cytokines, immune response	rs2430561	USA	ca = 20, co = 57	*p* = 0.04	OM with RSV infection	Gentile et al., 2003
*IL10*	1	Interleukin 10 (IL-10)	Cytokines, immune response	rs1554286, rs1800872, rs1800890, rs1800893, rs1800896, rs3024509	USA	142 families	p(ht) = 0.012, p(ht) = 0.039, p(ht) = 0.017, p(ht) = 0.017, p(ht) = 0.017, *p* = 0.040	RAOM/COME	Sale et al., 2011
				rs1800896	Netherlands	ca = 348, co = 463	*p* = 0.01	Protective for AOM after PV	Emonts et al., 2007
				rs1800871	Greece	ca = 96, c = nil	*p* < 0.0001	AOM	Ilia et al., 2014
				rs1800896, rs1800871, rs1800872	USA	ca = 102, co = 98	*p* = 0.005 *p* = 0.05 *p* = 0.05	OM followed RSV/RV	
*IL1A*	2	Interleukin I- (IL-1 alpha)	Cytokines, immune response	rs1800587	Finland	ca = 63, co = 400	*p* = 0.03	RAOM	Joki-Erkkila et al., 2002
*IL1B*	2	Interleukin 1-β (IL-1β)	Cytokines, immune response	rs16944	USA	ca = 653, co = nil	OR = 1.35	OM (prone)	Nokso-Koivisto et al., 2014
				rs1143634		ca = 104, co = 24	*p* = 0.02	AOM (inflammation)	
*IL6*	7	Interleukin 6 (IL–6)	Cytokines, immune response	rs1800795	Netherlands	ca = 347, co = 460	OR > 1.45; *p* = 0.02	AOM	Emonts et al., 2007
				rs1800795	USA	ca = 68, co = 145	*p* < 0.01	RAOM	Revai et al., 2009
				rs1800795	USA	ca = 192, co = 192	*p* = 0.03	AOM	Patel et al., 2006
				rs1800795	USA	ca = 77, co = 80	*p* < 0.01	AOM	Nokso-Koivisto et al., 2014
*MBL2*	10	Mannose-binding lectins (MBL)	Immune response	rs11003125, rs1800450, rs1800451, rs5030737, rs7095891, rs7096206	Belgium	ca = 17, co = 172	OR(ht) = 2.9	AOM	Nuytinck et al., 2006
*mDNA*	mt	n/a	Mitochondrial DNA	p.Thr195Cys	Czech Republic	ca = 355	*p* = 0.032	AOM	Sale et al., 2011
*MUC2*	11	Mucin-2	Gel-forming mucin, lubrication, viscoelasticity	rs7396030	USA	142 families	*p* = 0.049	RAOM/COME	Sale et al., 2011
				rs7396030	USA	441 families	*p* = 0.022	RAOM/COME	
*MUC5AC*	11	Mucin-5AC	Gel-forming mucin, lubrication, viscoelasticity	MUC5AC (intronic)	USA	ca = 40, co = 40	*p* = 0.025	RAOM/COME	Ubell et al., 2010
*MUC5B*	11	Mucin-5B	Gel-forming mucin, lubrication, viscoelasticity	rs4963049	USA	ca = 102, co = 83	*p* = 0.033	COME	MacArthur et al., 2014
				rs2075859	USA	142 families	*p* = 0.041	RAOM/COME	Sale et al., 2011
				rs2735733	USA	142 families	*p* = 0.02	RAOM/COME	Sale et al., 2011
*PAI1*	7	Plasminogen activator inhibitor-1 (PAI1)	Inflammation	rs1799889	Netherlands	ca = 226, co = 122	*p* = 0.02	RAOM	Emonts et al., 2007
*SCN1B*	19	Sodium channel sub-unit β1 (SCN_1_β)	Ion channel binding, voltage-gated ion channel activity	rs8100085	USA	ca = 142 families	*p* = 0.013	RAOM/COME	Sale et al., 2011
*SFTPA1*	10	SFTPA1	Phospho-lipoproteins, surfactant	sa4-1a haplotype	Finland	ca = 147,co = 278	p(ht) = 0.03	RAOM	Ramet et al., 2001
*SFTPD*	10	SFTPD	Phospho-lipoproteins, surfactant	RS1051246	USA	142 families	*p* = 0.039	RAOM/COM	Sale et al., 2011
*SLC11A1*	2	Solute carrier family 11 member (SLC11A1)	Transporter, pathogen clearance	rs2276631, rs02695343, rs34448891, rs3731865	Australia	ca = 531 families, co = 660	p(ht) = 0.008	OM Proneness	Rye et al., 2013
*SMAD2*	18	SMAD2	Transcriptional modulator activated by TGF-beta	rs1792658	Australia	ca = 434 families, co = 561	*p* = 0.038	RAOM/COME	Rye et al., 2011
*SMAD4*	18	SMAD4	Transcriptional modulator activated by BMP	rs10502913	Australia	ca = 434 families, co = 561	*p* = 0.048	RAOM/COME	Rye et al., 2011
*TGFB1*	19	Transforming growth factor beta 1 (TGF-β1)	Antigen binding, immune response	rs1982073	Greece	ca = 96	*p* = 0.002	AOM	Ilia et al., 2014
*TLR2*	4	Toll-like receptor 2 (TLR2)	Inflammation, initiators of innate immunity system	rs5743708	Serbia	ca = 85, co-100	Significantly high	COME	Lee H.Y. et al., 2008
*TLR4*	9	Toll-like receptor 4 (TLR4)	Inflammation, initiators of innate immunity system	rs1800896, rs1800871, rs1800872	USA	ca = 172, co = 83	*p* = 0.005 *p* = 0.05 *p* = 0.05	AOM	Ilia et al., 2014
				rs11788318, rs4837494, rs10116253, rs1927914, rs1554973	USA	ca = 102, co = 83	*p* = 0.008, *p* = 0.031, *p* = 0.007, *p* = 0.023, *p* = 0.021	COME	MacArthur et al., 2014
				rs10116253, rs12377632, rs22770146, rs5030717	USA	142 families	p (ht) = 0.025, p(ht) = 0.014, *p* = 0.026, p(ht) = 0.017	COME/RAOM	Sale et al., 2011
				rs5030717, rs1329060, rs1329057	Finland	ca = 624, co = 778 1,269 trios 403 families ca = 100, co = 104	OR 1.33, *p* = 0.003 OR 1.33, *p* = 0.002 OR 1.29, *p* = 0.003	COME/RAOM	Hafren et al., 2015
*TNFA*	6	Tumor necrosis factor α (TNF α)	Cytokines, immune response	rs1800629	USA	ca = 192, co = 192	*p* = 0.05	AOM	Emonts et al., 2007
						ca = 222, co = 120			
					Netherlands		*p* = 0.07	AOM	Revai et al., 2009
				rs1800750					
						ca = 68, co = 145			
							OR = 1.42	RAOM	Revai et al., 2009
				rs1800750	USA				

**Ca, cases; Co, controls; OR, odds ratio; p, p-value; LOD, logarithm of the odds.**

### Early Studies

The genetic contribution to OM susceptibility became evident in the 1980s after several studies showed that the prevalence of OM was disproportionately high in some ethnicities (native Americans and Australian aborigines) and relatively low in individuals of African ancestry (Clements, 1968; Bhutta, 2015). A surveillance study on ear and nasopharyngeal carriage was conducted among remote Australian aboriginal communities in 2013 and found that 50% of young children (mean age 13 months) had OME, 37% had AOM, and 12% had CSOM (Leach et al., 2016). Today, CSOM continues to be strongly implicated in the prevalence of hearing and learning disorders in Australian aboriginal communities (Morris, 1998).

One of the earliest genetic studies on OM, conducted in 1983, analyzed the blood groups (ABO) in a cohort of 610 children with chronic otitis media with effusion (COME) and concluded that blood group “A” was a genetic risk factor for OM based on their observation of its higher prevalence in children with COME as compared to non-affected children (Mortensen et al., 1983). Later studies have shown that human leukocyte antigen (HLA) 2 and HLA3 are strongly associated with AOM, while patients with COME have a lower frequency of HLA2 (Kalm et al., 1991, 1994). The heritability and genetic components of time with and the number of episodes with OME and AOM during the first 2 years of life were also investigated in a twin and a triplet study in 1999 and found a strong association between the duration or the number of episodes of OM and genetic makeup (Casselbrant et al., 1999).

The contribution of genetics to OM susceptibility is supported by studies reporting a higher incidence of OM in children with chromosomal abnormalities. For example, the prevalence of OME in children with Down syndrome approaches 38% (Austeng et al., 2013). Genes present on chromosome 21 in combination with craniofacial defects such as midfacial hypoplasia, short palate, and Eustachian tube dysfunction (Shibahara and Sando, 1989) and defects of the immune system (Ram and Chinen, 2011) observed in children with Down syndrome may contribute to their increased risk of OM. *Ets1* gene, encoding a proto-oncogene, has been recently associated with craniofacial abnormalities and OM in a mouse study (see section Mouse and Mouse-to-Man Studies) (Carpinelli et al., 2015). In humans, the *ETS2* gene that also belongs to the proto-oncogene gene family is present on chromosome 21 and may contribute to OM susceptibility in Down syndrome.

Several studies conducted on cohorts with Turner syndrome, a genetic disorder of partial or complete loss of chromosome X in females, described a highly variable (ranging from 9.1 to 91%) incidence of AOM (Sculerati et al., 1990; Bois et al., 2018). While the karyotype analysis did not reveal any significantly high-risk subgroup, females with Turner syndrome also have greater prevalence and longer duration of middle ear pathologies (Gawron et al., 2008; Bois et al., 2018). These findings implicate some of the X chromosome genes in middle ear development, function, or health.

### Mouse and Mouse-to-Man Studies

The development and the phenotyping of transgenic and knockout mouse models in the last 30 years have significantly helped to identify several genes and genetic variations that confer susceptibility to OM in mice. Most of these mouse models spontaneously develop OM; studying their ear morphology and function provided insights into the disease pathophysiology at a molecular level. For instance, Eriksson et al. (2006) showed that plasminogen (*Plg*)-deficient mice spontaneously develop chronic OM by 18 weeks of age. Plasmin, the active serine proteinase enzyme form of PLG, is mainly involved in the dissociation of fibrin clots and promotes the degradation of the extracellular matrix (Ayon-Nunez et al., 2018). Plasmin plays a critical role in several cellular processes, including wound healing, immunity, tissue remodeling, inflammation, and cell migration (Tefs et al., 2006). Recent studies have shown that certain bacteria possess plasminogen-binding adhesions on their cell surface to exploit the fibrinolytic system, facilitating bacterial uptake and invasion (Raymond and Djordjevic, 2015; Ayon-Nunez et al., 2018).

The role of transcription factors in OM pathology became apparent through the studies of mutant mice lacking *Eya4*, *Evi1*, *Tgif*, *Ets1*, and *Fli1* genes (Hardisty-Hughes et al., 2006; Parkinson et al., 2006; Depreux et al., 2008; Tateossian et al., 2013; Carpinelli et al., 2015). Mice lacking *Eya4* have Eustachian tube dysfunction, leading to an increased incidence of OME and hearing impairment (Depreux et al., 2008). Variants in *Evi1* in *Junbo* mice have been shown to cause susceptibility to CSOM. *Junbo* mice accumulate middle ear effusions and develop hypoxia, inflammation, and thickening of the mucoperiosteum (Parkinson et al., 2006; Bhutta et al., 2014). Later studies have shown that the loss of BPIFA1, one of the most abundant secretory proteins in the upper respiratory tract (Musa et al., 2012), exacerbates the severity of OM in *Junbo* mice. While *Bpifa1* mutant mice did not show any OM susceptibility, the deletion of *Bpifa1* in mice carrying *Evi1 Junbo* variant leads to the thickening of the middle ear mucosa and an increase of collagen deposition (Mulay et al., 2018). Loss of *Tgif1*, which encodes for TGIF1, results in OME accompanied by the thickening of the middle ear epithelial lining, an increase of goblet cell population, elevated levels of TNF-α and IL-1β in ear fluids, and conductive hearing loss in mice (Tateossian et al., 2013). Similarly, haploinsufficiency for *Ets1* and *Fli1* in mice results in craniofacial abnormalities, including a smaller middle ear cavity and fusion of ossicles to the walls of the middle ear (Carpinelli et al., 2015). Furthermore, *Fli1*^±^ and *Ets1*^±^ double-mutant mice have hearing impairment and their middle ear mucosa is infiltrated by proinflammatory cells, leading to OM (Carpinelli et al., 2015).

Hardisty et al. (2003) showed that *Jeff* mutant mice carrying a *Fbxo11* variant have craniofacial abnormalities, elevated hearing thresholds, and middle ear effusion. Defects in the bulla cavitation were observed in *Fbxo11* mutant mice, which ultimately result in middle ear adhesions and soft tissue mineralization of the bony anatomy (Del-Pozo et al., 2019). Using N-ethyl-N-nitrosourea mutagenesis, Crompton et al. (2017) showed that the pathogenic variant, p.Leu972Pro, also known as *edison* variant, in the *Nischarin* (*Nisch*) gene leads to mild craniofacial defects, spontaneous OM by 20 weeks, and progressive hearing loss. Recent studies have reported the association of *TGIF1* and *NISCH* loci as potential risk areas for OM in humans (Bhutta et al., 2017), thus supporting the relevance of knowledge obtained from mouse models to the pathophysiology of OM in humans.

Finally, *Eda* and *Edar* transcription factors mutant mice (*Eda*^*Ta*^ and *Edar*^*dlJ/dlJ*^) also developed chronic rhinitis and OM (Azar et al., 2016). In these mutants, the nasopharyngeal glandular epithelium fails to develop, which leads to the loss of lysozyme secretion, the reduction of mucociliary clearance, and the overgrowth of commensal bacteria. The spread of nasal *S. aureus* in *Eda*^*Ta*^ mice and of *Escherichia coli* in *Edar*^*dlJ/dlJ*^ mice into the middle ear bulla potentially triggers inflammation and OM (Azar et al., 2016). A non-exhaustive list of the most common loci associated with OM in mouse is presented in Table 1.

### Human Candidate Gene-Based and Genome-Wide Association Studies

While early candidate gene-based OM studies have been done mostly on Caucasian patients with recurrent AOM and chronic OME (see section Early Studies), more recent genetic studies have been focusing on ethnic groups or communities for which marriages within the families are relatively common (e.g., indigenous Filipino community or Pakistani families) (Santos-Cortez et al., 2015, 2018). These studies have unveiled several novel genes and variants that confer susceptibility to familial OM (see section OM Susceptibility, Inbreeding, and Whole-Exome Sequencing): *A2ML1* and *FUT2* (Santos-Cortez et al., 2015, 2018).

### Candidate Gene-Based Studies

Many variants in the genome have been associated with infectious diseases (Klebanov, 2018). In some instances, the clinical features and the biological mechanisms – such as immune response, inflammation, bacterial adhesion, viral infection, and mucociliary clearance – involved in those infectious diseases are compatible with the mechanisms involved during an episode of OM, thus marking the genes known for these disorders as prime candidates for OM susceptibility and recurrence. Candidate gene-based studies on OM have mainly involved genes associated with innate immunity and inflammation (Sale et al., 2011). Those studies have been performed on cohorts from all over the world including US, Finland, Australia, Netherlands, Greece, and Belgium and have identified over 100 alleles that confer susceptibility to various forms of OM (see Table 2 for the partial list of these alleles, genes, and associated clinical features). As evident from this non-exhaustive list, the identified genes belong to several different signaling cascades and developmental processes, including (a) immune response and inflammation (*MBL2*, *TLR2*, *TLR4*, *CD14*, *FCGR2A*, *TGFB1*, and *PAI1*) (Nuytinck et al., 2006; Wiertsema et al., 2006a, b; Emonts et al., 2007; Lee Y.C. et al., 2008; Ilia et al., 2014; Hafren et al., 2015), (b) cytokines (*IL6*, *IL10*, *IL1A*, *IL1B*, *TNFA*, and *IFNG*) (Patel et al., 2006; Alper et al., 2009; Revai et al., 2009; Ilia et al., 2014), (c) tissue clearance (*SFPTA*, *SFTPA1*, *SFTPD*, *SLC11A1*, *MUC2*, *MUC5AC*, and *MUC5B*) (Ramet et al., 2001; Sale et al., 2011; Rye et al., 2013; MacArthur et al., 2014), (d) transcriptional modulation (*SMAD2* and *SMAD4*) (Rye et al., 2011), (e) chemosensitivity (*CX3CR1*) (Nokso-Koivisto et al., 2014), (f) protein modification (*CPT1A* and *FBXO11*) (Rye et al., 2011, 2012), and (g) channel activity (*SCN1B*) (Sale et al., 2011). Some of these genes, such as *FBXO11*, have been replicated in several independent studies, which further strengthen their role in susceptibility to OM (Segade et al., 2006; Rye et al., 2011).

### Genome-Wide Association Studies

Several genome-wide association studies (GWAS) have been performed to identify new common (frequency of 75% or greater) low-risk markers (OR < 1.5) associated with OM subtypes. The findings of five salient GWAS are summarized here. In the Western Australian Pregnancy Cohort (Raine) study, a cohort of 416 patients prone to OM and 1,075 normal subjects was analyzed for 2,524,817 SNPs. Although the initial analysis revealed some association, no SNP reached GWAS significance (*P* < 10^–8^) nor could be replicated both in the Australian or US cohorts (Rye et al., 2012; Allen et al., 2014). Intriguingly, the GWAS of the Minnesota and Pittsburg cohorts identified a SNP (rs10497394 on chromosome 2) that showed a significant association (GWAS discovery *P* = 1.30 × 10^–5^, independent otitis media population *P*_*meta*_ = 1.52 × 10^–8^) with susceptibility to either chronic OME or recurrent AOM (Allen et al., 2013). Finally, in a Finnish cohort (829 affected children and 2,118 randomly selected controls), the variants rs16974263 (GWAS discovery *P* = 1.77 × 10^–7^, sub-phenotype analysis *P*_*meta*_ = 2.92 × 10^–8^), rs268662 (*P* = 1.564 × 10^–6^), and rs4150992 (*P* = 3.37 × 10^–6^) were the most significant variants associated with COME (Einarsdottir et al., 2016). In van Ingen et al. (2016) performed GWAS on a cohort of AOM children of European descent and reported a statistically significant association at 6q25.3 locus (rs2932989, *P*_*meta*_ = 2.15 × 10^–9^). This study further demonstrated that the associated variants are correlated with the methylation status (cg05678571, *p* = 1.43 × 10^–6^) and expression levels (*p* = 9.3 × 10^–5^) of the *FNDC1* gene. Also, an independent GWAS study on more than 200,000 individuals of European ancestry reported 14 genomic regions, including *FUT2* (*p*-value: 3.51 × 10^–30^), *TBX1* (1.17 × 10^–19^), *HLA-DRB1* (rs4329147, 9.55 × 10^–12^), *ABO* (3.67 × 10^–11^), *EFEMP1* (1.47 × 10^–10^), *AUTS2* (3.75 × 10^–9^), *CDHR3* (5.40 × 10^–9^), *BSN* (1.56 × 10^–8^), and *PLG* (3.78 × 10^–8^), that were significantly associated with childhood ear infection (Tian et al., 2017), further highlighting the contribution of genetic factors responsible for OM in humans.

### OM Susceptibility, Inbreeding, and Whole-Exome Sequencing

#### A2ML1

In a large consanguineous indigenous Filipino pedigree with a high frequency of OM, Santos-Cortez et al. (2015) showed, by whole-exome and Sanger sequencing, that an 8 bp duplication in the *A2ML1* gene (LOD score of 7.5) was associated with susceptibility to OM. The same duplication was found in a heterozygous or a homozygous fashion in three individuals (European American and Hispanic) of another cohort of 123 children prone to OM and absent in non-otitis-prone children and more than 62,000 next-generation sequences. The authors identified seven additional heterozygous *A2ML1* variants in patients of European American and Hispanic American origin with OM. *A2ML1* encodes alpha-2-macroglobulin-like protein 1 (A2ML1), a protein that traps proteinases and cleaves them. A follow-up study identified 16 additional A2ML1 variants in OM subjects in indigenous Filipino and Pakistani families and US probands (Larson et al., 2019). Based on the expression in the murine mucosal epithelium of the middle ear, it has been speculated that A2ML1 may have a protective function by regulating the proteases present in the middle ear cavity and may also regulate the desquamation of epidermis (Galliano et al., 2006). Recently, by 16S rRNA sequencing of the microbiota of the middle ear of an indigenous Filipino community prone to OM and segregating *A2ML1* variants, although not having a statistically significant difference between the cases and the controls, a taxonomic analysis revealed the relative abundance of the phyla *Fusobacteria* and *Bacteroidetes* and the genus *Fusobacterium* in *A2ML1* carriers compared to non-carriers (Santos-Cortez et al., 2016).

#### FUT2

In the same large consanguineous indigenous Filipino pedigree with high frequency of OM, partly due to variants in *A2ML1*, a subset of individuals were wild type for *A2ML1* but were prone to OM (Santos-Cortez et al., 2015). Further genetic analysis determined that the *FUT2* variant (p.Arg202^∗^, LOD score of 4.0) confer susceptibility to OM in those individuals (Santos-Cortez et al., 2018). Screening of DNA samples from 609 additional multi-ethnic families and simplex case subjects with OM by direct Sanger sequencing, linkage analysis, Fisher exact, and transmission disequilibrium tests revealed several other *FUT2* variants (p.Arg138Cys, p.Trp154^∗^, and p.Ala104Val) that confer susceptibility to OM (Santos-Cortez et al., 2018).

FUT enzymes are involved in the protein glycosylation pathway. FUTs transfer an L-fucose sugar derived from GDP-fucose (donor substrate) to a protein (acceptor substrate). The FUT family contains 13 members (FUT1–FUT13), and many of the FUTs are essential for the synthesis of blood group antigens. FUTs are single-pass type II membrane proteins, resident to the trans-Golgi, while the catalytic domain of FUT proteins resides in the lumen of the Golgi. In humans, *FUT1* and *FUT2* encode galactoside 2-L-fucosyltransferase, while *FUT3* encodes galactoside 3(4)-L-fucosyltransferase. FUT1 and FUT2 transfer L-fucose onto a β-D-galactosyl-(1→4)-N-acetyl-β-D-glucosaminyl derivative and create the oligosaccharide FuC-alpha [(1,2)Gal-beta-], also known as H antigen, which is a soluble precursor essential for the final step in the soluble A and B antigen synthesis pathway. FUT3 transfers L-fucose onto a β-D-galactosyl-(1→4)-N-acetyl-β-D-glucosaminyl derivative, or onto H antigen, in order to create blood group Lewis antigens.

*FUT1* and *FUT2* are differentially expressed in various cell types. For instance, *FUT1* expression is restricted to cells of mesodermal origin (for example, erythrocytes), and *FUT2* expression is being restricted to cells of endodermal origin (such as the middle ear mucosal cells). Therefore, A and B antigens will be expressed at the surface of red blood cells under the control of FUT1, while A and B antigens will be expressed at the surface of mucosal cells under the control of FUT2. Genetic variations in *FUT1* and *FUT2* naturally exist. Some *FUT1* and *FUT2* variants lead to non-functional enzymes, while certain variations in *FUT2* can also lead to a reduction of its expression (Santos-Cortez et al., 2018). For instance, the FUT1 p.Tyr154Cys variant ablates the functional activity of the catalytic domain, resulting in the absence of A, B, or H antigens at the surface of erythrocytes (also known as the Bombay phenotype). Similarly, the FUT2 p.Trp154^∗^ variant causes absence of A, B, or H antigens at the surface of mucosal cells (a.k.a. non-secretor status) (Domino et al., 2001a, b). The p.Trp154^∗^ variant of FUT2 is also responsible for the non-secretor phenotype in European and African populations (47 and 42%, respectively).

**FIGURE 1:**
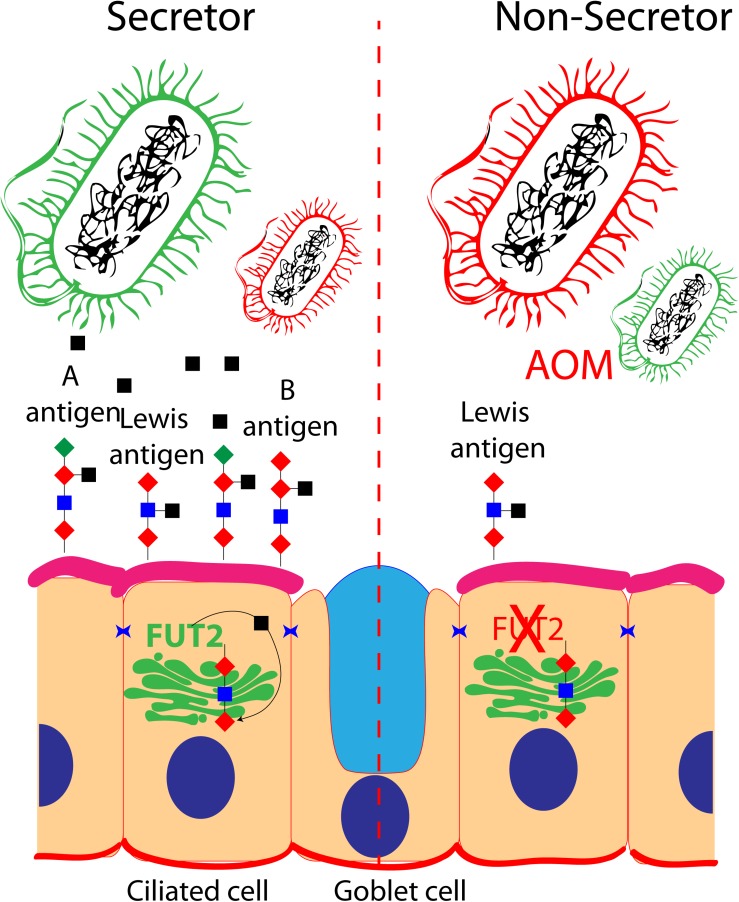
Role of *FUT2* in the regulation of blood group and Lewis antigens at the surface of ciliated cells of the middle ear mucosa: secretor and non-secretor status are illustrated. The imbalance of optimal bacteria (green) and pathogenic bacteria (red) is shown in a non-secretor individual that is prone to acute otitis media.

A, B, and H and Lewis antigens are known to serve as an energy source while also regulating the adhesion of bacteria to the cell surface (Ewald and Sumner, 2018; Figure 1). Intuitively, the different blood group antigens and their quantity at the surface of the cells of the mucosal epithelium of the middle ear would have an impact on the microbiota present in the middle ear cavity, and various blood types have been associated with OM (Wiesen et al., 2019). For instance, studies in a Finnish cohort reported a protective effect of blood type O against recurrent AOM, whereas blood type A was associated with increased risk for chronic OME (Wiesen et al., 2019). When tested *in vitro*, the four *FUT2* variants associated with OM (p.Ala104Val, p.Arg138Cys, p.Trp154^∗^, and p.Arg202^∗^) reduced the A antigen levels, while the two nonsense variants also reduced the FUT2 protein levels. Moreover, *Fut2* is transiently upregulated in the murine middle ear after inoculation with the non-typeable *H. influenza* (Santos-Cortez et al., 2018). It is speculated that the OM-associated *FUT2* variants are modifying the middle ear microbiome through the regulation of A antigen levels in the middle ear mucosa, thus conferring susceptibility to OM (Santos-Cortez et al., 2018).

While the microbial richness, structure, and composition differences were not statistically significant between the control individuals and the individuals prone to OM in the indigenous Filipino community segregating *FUT2* p.Arg202^∗^ variant, the individuals from a Colorado cohort prone to OM and carrier for the *FUT2* p.Trp154^∗^ variant had a relatively high abundance of *Lactobacillales* and *Gamma-proteobacteria* in their middle ears (Santos-Cortez et al., 2018). Further studies in animal models are necessary to fully understand the FUT2-associated OM mechanism.

## Conclusion

The current genetic and molecular data revealed the association of OM with deficits in each of the following mechanisms: (1) development of the middle ear cavity and Eustachian tube, (2) immune response, (3) bacterial adhesion and viral infection, (4) regulation of the extracellular matrix, and (5) clearance of the middle ear.

**FIGURE 2:**
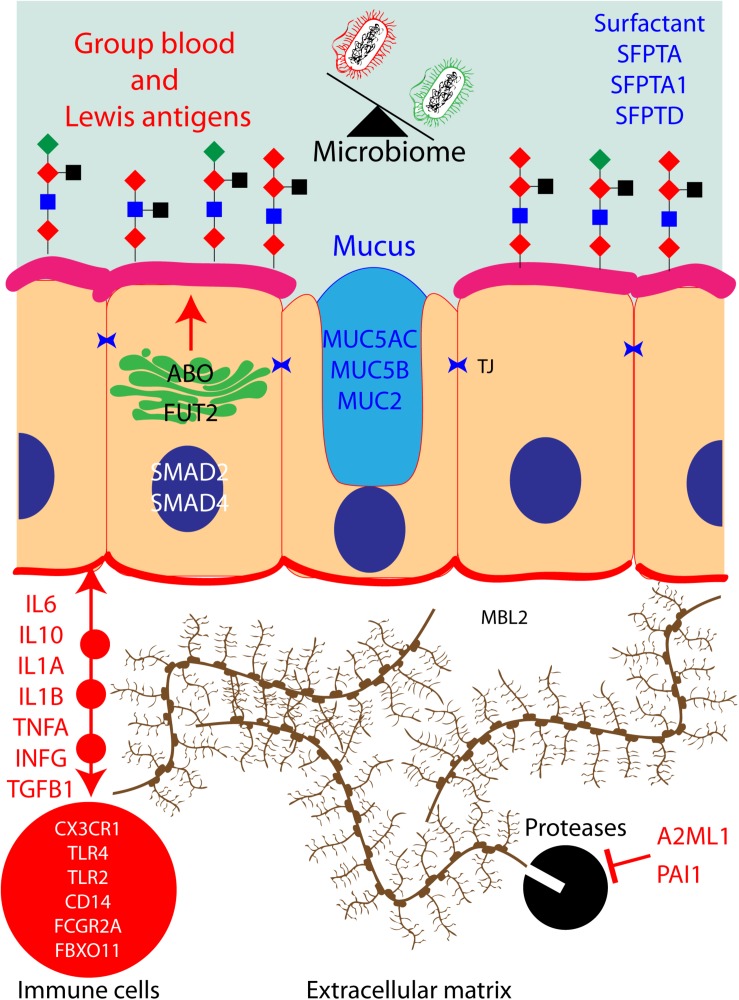
Protein pathways contributing to otitis media (OM) in human: hypothetical network of human proteins in the middle ear mucosa that have been associated with OM. When these proteins are dysfunctional due to OM-associated genetic variants, they could potentially have an impact on the microbiota of the middle ear cavity.

In Figure 2, we have attempted to build a network that encompasses most of the known human proteins that have been associated with OM and our hypothesis about the potential impact on the microbiome of the middle ear cavity when these proteins are dysfunctional due to OM-associated genetic variants. Briefly, ABO and FUT2 are localized in the Golgi apparatus of the cells of the middle ear mucosa; these two proteins together create blood group Lewis antigens, potential sources of energy for microbiome. These antigens also provide an adhesion platform for the microbiota. Similarly, goblet cells secrete MUC5AC, MUC5B, MUC2 SFPTA, SFPTA1, and SFPTD to form mucus and surfactants in the middle ear cavity. Bacteria are present in these secretions and are cleared from the middle ear cavity by ciliated cells in order to maintain a healthy microbiome. Cells from the middle ear also secrete interleukins, chemokines, interferons, and necrosis and growth factors in order to recruit immune cells in the extracellular matrix. These cells fight infection and eliminate dead cells. During the inflammatory stage, the extracellular matrix is remodeled by proteases to allow immune cell infiltration. Those proteases are regulated and inhibited by A2ML1 and PAI1. A pathogenic genetic variation in any of those genes would eventually lead to middle ear infection and OM.

Future studies of the enrichment of certain microbiota in individuals with specific genetic variants may eventually help in identifying patients before chronic OM sets in or in devising a patient-specific treatment paradigm in the future.

## Author Contributions

AG, SA, AI, IA, SR, and ZA wrote the draft and finalized it.

## Conflict of Interest

The authors declare that the research was conducted in the absence of any commercial or financial relationships that could be construed as a potential conflict of interest.
